# Enhancing effect of bromovinyldeoxyuridine on antitumour activity of 5-fluorouracil in mice bearing MOPC-315 plasmacytomas.

**DOI:** 10.1038/bjc.1986.250

**Published:** 1986-11

**Authors:** S. Ben-Efraim, S. Shoval, E. de Clercq


*Br. J. Cancer* (1986), **54**, 847–851

### Short Communication

### Enhancing effect of bromovinyldeoxyuridine on antitumour activity of 5-fluorouracil in mice bearing MOPC-315 plasmacytomas

S. Ben-Efraim[1], S. Shoval[1] & E. de Clercq[2]

[1]*Department of Human Microbiology, Sackler School of Medicine, Tel-Aviv University, Tel-Aviv 69978, Israel; and* [2]*Rega Institute for Medical Research, Katholieke Universiteit Leuven, B-3000 Leuven, Belgium.*

It has recently been shown (Desgranges *et al.*, 1986) that bromovinyluracil [BVU: (E)-5-(2-bromovinyl)uracil] increases the antitumour effect of 5-fluorouracil (5-FU) in DBA/2 mice inoculated with P388 leukaemia cells, most likely by inhibiting 5-FU degradation *in vivo*. The inhibitory effect is apparently due to the effect of BVU on dihydrothymine dehydrogenase, the rate-limiting enzyme in the catabolism of pyrimidines. It has also been reported that bromovinyldeoxyuridine [BVDU; (E)-5-(2-bromovinyl)-2′-deoxyuridine] is rapidly converted to BVU *in vivo* (Desgranges *et al.*, 1984) and induces the same synergistic antitumour effect with 5-FU as observed upon injection of BVU (Desgranges *et al.*, 1986). BVDU itself is an antiviral compound with great promise for the treatment of herpes simplex virus (type 1) and varicella-zoster virus infections (de Clercq & Walker, 1984).

We have shown (Ben-Efraim *et al.*, 1986) that treatment of MOPC-315 plasmacytoma tumour cells for 1 h with a minimal dose of 0.1 $\mu$g 5-FU/$10^7$ cells caused marked reduction in incorporation of (methyl)$^3$H thymidine (up to 68% of the degree of thymidine incorporation in control untreated cells). No attempts were made to determine the effect of extending the length of the 5-FU treatment interval on tumour cells. In view of results obtained with other tumour lines (Drewinko & Yang, 1985), it might be that extending the length of 5-FU treatment of tumour cells will result in more marked inhibition of thymidine incorporation and possibly in reduction of the minimal effective dose of 5-FU.

The data indicating tumouricidal effect *in vitro* of 5-FU on MOPC-315 tumour cells prompted us to evaluate the effect of 5-FU therapy in mice bearing MOPC-315 plasmacytoma tumours. We found (Ben-Efraim *et al.*, 1986) that a single injection of 5-FU at 200 mg kg$^{-1}$ in BALB/c mice bearing large MOPC-315 plasmacytomas induced transient regression of the tumours. At doses <200 mg kg$^{-1}$, 5-FU was without effect (Ben-Efraim *et al.*, 1986). In view of the inhibitory effect of BVDU (via BVU) on the degradation of 5-FU (Desgranges *et al.*, 1986), it appeared of interest to determine whether low doses of 5-FU which did not affect development of established MOPC-315 plasmacytoma tumours alone, could be more effective when administered in combination with BVDU.

The MOPC-315 myeloma cell line employed in this study grows preferentially in the mouse BALB/c strain and is characterized by its ability to secrete anti-TNP trinitrophenyl) IgA$\lambda_2$ immunoglobulin (Eisen *et al.*, 1968). Subcutaneous inoculation of $1 \times 10^5$ MOPC-315 tumour cells invariably induces formation of local tumour which reach a size of ~15.0 mm on the 11th day after inoculation and death within 20 days. An *in vitro* line of MOPC-315 tumour cells, adapted to growth in culture (Yaniv *et al.*, 1978), was maintained in RPMI 1640 medium (GIBCO, NY, USA), supplemented with 100 U ml$^{-1}$ penicillin, 100 $\mu$g ml$^{-1}$ streptomycin, 2 mmol ml$^{-1}$ L-glutamine, and 10% foetal calf serum. The MOPC-315 cell line was used for evaluating the *in vitro* effects of BVDU and 5-FU on (methyl)$^3$H thymidine (Nuclear Research Centre, Negev, Israel; 1.0 $\mu$Ci/50 $\mu$l/culture) incorporation in tumour cells. BVDU was synthesized at the Rega Institute for Medical Research Leuven, Belgium following a described procedure (Jones *et al.*, 1979). 5-FU was kindly donated by Abic, Ramat-Gan, Israel. The effect of BVDU and 5-FU on MOPC-315 tumour cells *in vitro* were monitored in incubating $1 \times 10^7$ cells with varying compound concentrations for 1 h at 37°C, washing the cells with serum-free medium and further incubating the cell cultures as described (Bocian *et al.*, 1984). For the experiments *in vivo*, groups of ten BALB/c mice

Correspondence: S. Ben-Efraim.
Received 21 April 1986; and in revised form, 2 July 1986.

© The Macmillan Press Ltd., 1986

(8–12 weeks old) were inoculated s.c. with $1 \times 10^5$ viable MOPC-315 tumour cells. BVDU (200 $\mu$mol – 69.5 mg kg$^{-1}$) was injected i.p. in a volume of 0.5 ml on the 11th day after tumour cell inoculation, followed 1 h later by a single i.p. injection of varying doses of 5-FU in 0.5 ml. Untreated inoculated mice and mice treated with either BVDU or 5-FU alone served as controls. The mice were observed for 60 days for tumour development and death.

The significance of differences between groups treated with 5-FU alone and corresponding groups treated with 5-FU plus BVDU was calculated by the double tailed Mann-Whitney *U* test. The rate of tumour development in various groups as indicated by measurements of tumour sizes was compared till the 19th day after inoculation. Until this day, all of the inoculated mice were still alive in the compared groups. Differences were considered significant when $P < 0.05$. The therapeutic index (TI) was calculated as ratio of maximum tolerated dose (MLD) to minimum effective dose (MED). According to our previously reported data (Ben-Efraim *et al.*, 1986) the MLD for 5-FU was 250 mg kg$^{-1}$ body weight. The MED was taken as the lowest quantity of 5-FU which induced significant transient regression of large MOPC-315 tumours. Injection of 5-FU, 100 mg kg$^{-1}$ body wt plus BVDU (69.5 mg kg$^{-1}$) in normal, noninoculated mice did not affect the survival of animals and did not cause appreciable loss of weight.

Exposure of MOPC-315 tumour cells *in vitro* to different concentrations of BVDU (0.5 to 100 $\mu$g $10^{-7}$ cells) for 1 h did not affect the rate of thymidine incorporation into the tumour cells. In keeping with previous results (Ben-Efraim *et al.*, 1986), treatment of MOPC-315 tumour cells with 5-FU markedly reduced the extent of thymidine incorporation into the cells (Table I). Treatment with BVDU alone (69.5 mg kg$^{-1}$) or 5-FU alone (12.5 to 100 mg kg$^{-1}$), did not affect the growth of MOPC-315 tumour *in vivo*. If however, 5-FU treatment was combined with BVDU treatment, 5-FU at doses ranging from 25 to 100 mg kg$^{-1}$, caused a transient regression of MOPC-315 tumours and prolongation of survival time. The kinetics of tumour development are presented in Figures 1a, b and the rates of survival are shown in Figures 2a, b. The therapeutic index (TI) was 2.5 for mice treated with 5-FU alone (250 mg kg$^{-1}$ MLD/100 mg kg$^{-1}$ MED) and 20.0 for mice treated with a combination of 5-FU plus BVDU (250 mg kg$^{-1}$ MLD/12.5 mg kg$^{-1}$ MED).

Our findings indicate that BVDU has a potentiating effect on the antitumour activity of 5-FU in mice bearing large progressively growing MOPC-315 tumours. These results are in agreement with previously reported data (Desgranges *et al.*, 1986) on the synergistic antitumour activity of BVDU and 5-FU in DBA/2 mice inoculated with P388 leukaemia cells. BVDU alone does not affect MOPC-315 tumour cells either *in vitro* or *in vivo*. Since BVDU is readily converted *in vivo* to BVU (Desgranges *et al.*, 1984) and since BVU is capable of inhibiting the degradation of 5-FU *in vivo* (Desgranges *et al.*, 1986), one may postulate that the increased antitumour activity of combined BVDU and 5-FU therapy in the MOPC-315 model is due to a decrease in the degradation of 5-FU, and hence greater bio-availability of the compound.

Under the experimental conditions used (i.e. single doses of both BVDU and 5-FU), only a transient regression of MOPC-315 tumours was observed. It would seem imperative to examine whether a more intensive treatment regimen, i.e. higher doses of BVDU and/or repeated administrations of BVDU and 5-FU, may yield a more definitive prolonged or even definitive regression of MOPC-315 tumours. In any case, the combination of 5-FU and BVDU is an interesting lead that should be further pursued as a therapeutic modality in the treatment of cancer. Improvement in effectiveness of 5-FU therapy may be of importance in view of the doubts expressed (Drewinko & Yang, 1985) on the efficacy of therapy with this drug alone.

**Table I** Effect of bromovinyldeoxyuridine (BVDU) and 5-fluorouracil (5-FU) on [methyl-H]thymidine incorporation in MOPC-315 tumour cells *in vitro*.

| *Treatment*[a] | | *[methyl-$^3$H]thymidine incorporation*[b] | |
|---|---|---|---|
| *Compound* | *μg $10^{-7}$ cells* | *cpm ± s.e.* | *% of control* |
| None | —— | 220,064 ± 12,994 | —— |
| BVDU | 0.5 | 291,128 ± 5,030 | 132.2 |
| | 5.0 | 219,924 ± 3,126 | 99.9 |
| | 15.0 | 217,218 ± 3,604 | 98.7 |
| | 30.0 | 196,562 ± 3,394 | 89.3 |
| | 100.0 | 229,560 ± 5,943 | 104.3 |
| None | —— | 178,044 ± 3,933 | —— |
| 5-FU | 3.0 | 79,837 ± 15,360 | 44.7 |
| | 10.0 | 42,312 ± 5,596 | 23.7 |
| | 30.0 | 57,829 ± 3,936 | 32.4 |

[a]MOPC-315 tumour cells were incubated for 1 h with the compound at 37°C and washed 3 times before culturing; tumour cells incubated in medium alone were used as controls; [b]Tumour cells were cultured for 72 h; [methyl-$^3$H]thymidine (1 $\mu$Ci/culture) was added for the last 6 h of incubation; $2 \times 10^4$ tumour cells/culture; cpm represent means (±s.e.) for 16 parallel samples; % of thymidine incorporation is expressed as function of the corresponding control.

**Figure 1** (a) Effect of treatment with bromovinyldeoxyuridine (BVDU) and 5-fluorouracil (5-FU) on the development of MOPC-315 tumours.

1 (— — —) inoculated, untreated mice.
2 (··········) inoculated, 5-FU 100 (mg kg$^{-1}$).
3 (- - - -) inoculated, 5-FU 100 (mg kg$^{-1}$)+BVDU 69.5 (mg kg$^{-1}$).
4 (------) inoculated, 5-FU 50 (mg kg$^{-1}$).
5 (—— - —) inoculated, 5-FU 50 (mg kg$^{-1}$)+BVDU 69.5 (mg kg$^{-1}$).
6 (———) inoculated, BVDU 69.5 (mg kg$^{-1}$).

Significant differences ($P<0.05$) were found between groups 4 and 5 on days 13–19 after inoculation.

(b) Effect of treatment with bromovinyldeoxyuridine (BVDU) and 5-fluorouracil (5-FU) on the development of MOPC-315 tumours.

1 (— — —) inoculated, untreated mice.
2 (··········) inoculated, 5-FU 25 (mg kg$^{-1}$).
3 (- - - -) inoculated, 5-FU 25 (mg kg$^{-1}$)+BVDU 69.5 (mg kg$^{-1}$).
4 (------) inoculated, 5-FU 12.5 (mg kg$^{-1}$).
5 (—— - —) inoculated, 5-FU 12.5 (mg kg$^{-1}$)+BVDU 69.5 (mg kg$^{-1}$).

Significant differences ($P<0.05$) were found between groups: 2 *vs*. 3: days 13–19 after inoculation; 4 *vs*. 5: days 13–16 after inoculation.

**Figure 2** (a) Survival times after inoculation with MOPC-315 tumour cells and treatment with bromovinyldeoxyuridine (BVDU) and 5-fluorouracil (5-FU).

1 (— — —) inoculated, untreated mice.
2 (··········) inoculated, 5-FU 100 ($mg\,kg^{-1}$).
3 (- - - -) inoculated, 5-FU 100 ($mg\,kg^{-1}$)+BVDU 69.5 ($mg\,kg^{-1}$).
4 (------) inoculated, 5-FU 50 ($mg\,kg^{-1}$).
5 (—·—·) inoculated, 5-FU 50 ($mg\,kg^{-1}$)+BVDU 69.5 ($mg\,kg^{-1}$).
6 (———) inoculated, BVDU 69.5 ($mg\,kg^{-1}$).

(b) Survival times after inoculation with MOPC-315 tumour cells and treatment with bromovinyldeoxyuridine (BVDU) and 5-fluorouracil (5-FU).

1 (— — —) inoculated, untreated mice.
2 (··········) inoculated, 5-FU 25 ($mg\,kg^{-1}$).
3 (- - - -) inoculated, 5-FU 25 ($mg\,kg^{-1}$)+BVDU 69.5 ($mg\,kg^{-1}$).
4 (------) inoculated, 5-FU 12.5 ($mg\,kg^{-1}$).
5 (—·—·) inoculated, 5-FU 12.5 ($mg\,kg^{-1}$)+BVDU 69.5 ($mg\,kg^{-1}$).

We thank Abic, Israel for the generous supply of 5-fluorouracil. This work was supported in part by an endowment bequeathed by Meir and Rebecca Heinik in memory of their son Joseph Heinrichson.

### References

BEN-EFRAIM, S., SHOVAL, S. & OPHIR, R. (1986). The difference between 5-fluorouracil and melphalan in their ability to promote antitumor immune response against MOPC-315 plasmacytoma. *Cancer Immunol. Immunother.*, **22,** 43.

BOCIAN, R.C., BEN-EFRAIM, S., DRAY, S. & MOKYR, M.B. (1964). Melphalan-mediated potentiation of antitumor immune responsiveness of immunosuppressed spleen cells from mice bearing a large MOPC-315 tumor. *Cancer Immunol. Immunother.*, **18,** 41.

DE CLERCQ, E. & WALKER, R.T. (1984). Synthesis and antiviral properties of 5-vinylpyrimidine nucleoside analogs. *Pharmac. Ther.* **26,** 1.

DESGRANGES, C., RAZAKA, G., DROUILLET, F., BRICAUD, H., HERDEWIJN, P. & DE CLERCK, E. (1984). Regeneration of the antiviral drug (*E*)-5-(2-bromovinyl)-2′-deoxyuridine *in vivo. Nucleic Acid Res.*, **12,** 2081.

DESGRANGES, C., RAZAKA, G., DE CLERCQ, E. & 4 others (1986). Effect of (*E*)-5-(2-bromovinyl) uracil on the catabolism and antitumor activity of 5-fluorouracil. *Cancer Res.*, **46,** 1094.

DREWINKO, B. & YANG, L.Y. (1985). Cellular basis for the inefficacy of 5-FU in human colon carcinoma. *Cancer Treat. Rep.*, **69,** 1391.

EISEN, H.N., SIMMS, E.S. & POTTER, M. (1968). Mouse myeloma proteins with antihapten antibody activity. The protein produced by plasma cell tumor MOPC-315. *Biochemistry,* **7,** 4126.

JONES, A.S., VERHELST, G. & WALKER, R.T. (1979). The synthesis of the potent anti-herpes virus agent, (*E*)-5-(2-bromovinyl)-2′-deoxyuridine and related compounds. *Tetrahedron Letters,* **20,** 4415.

YANIV, A., GAZIT, A., DVIR, M., GUTHMANN, D. & EYLAN, E. (1978). Adaptation of murine MOPC-315 myeloma cells in growth *in vitro* and further characterization of their C-type viruses. *Eur. J. Cancer,* **14,** 771.


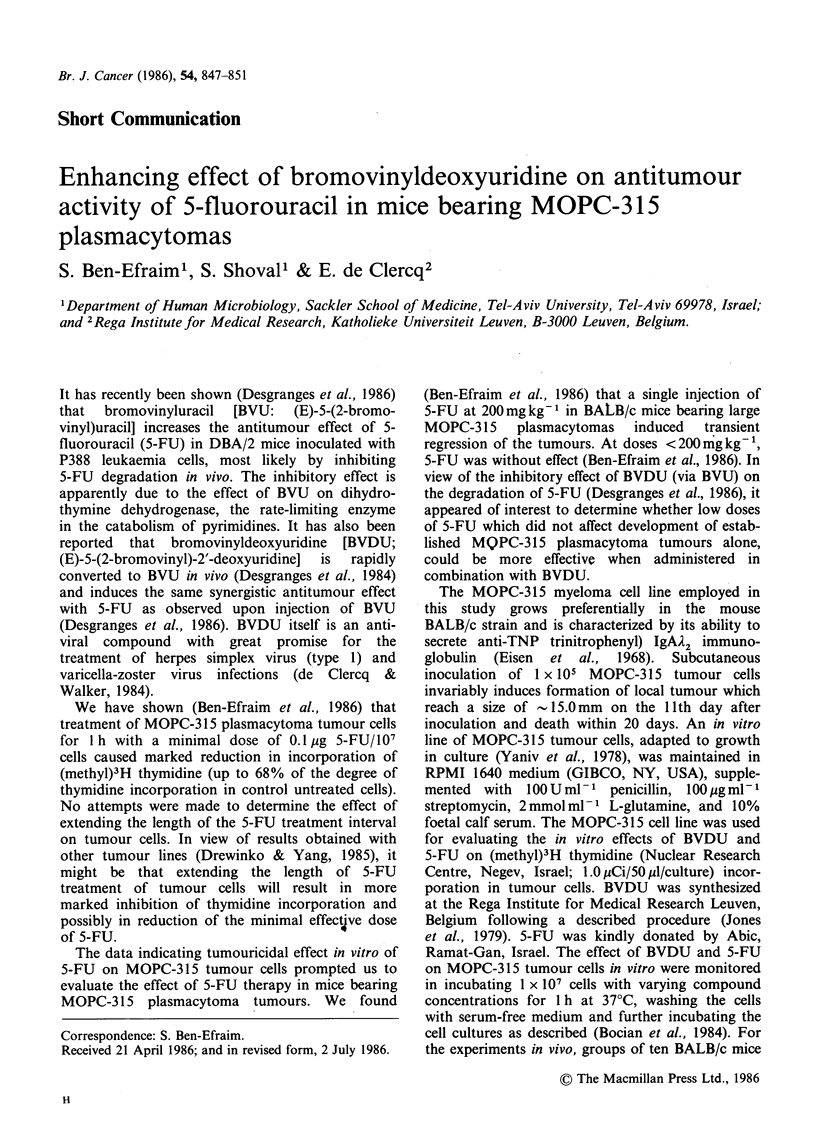

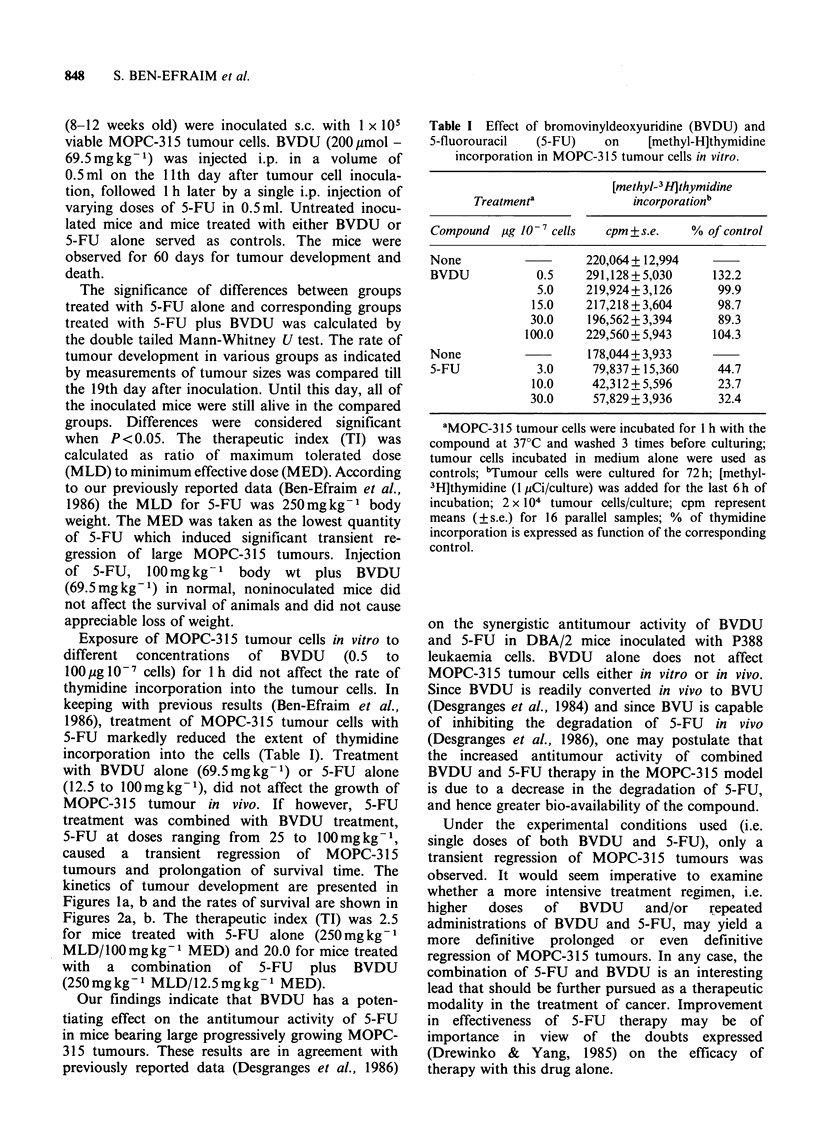

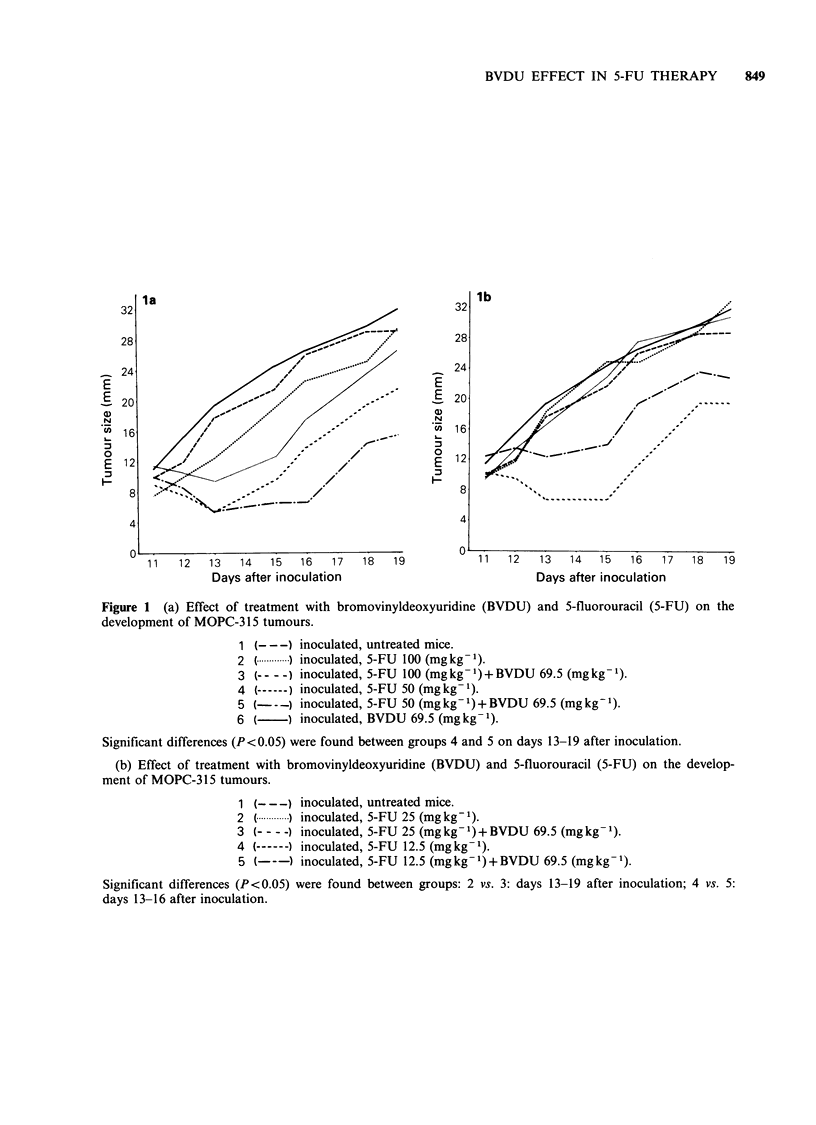

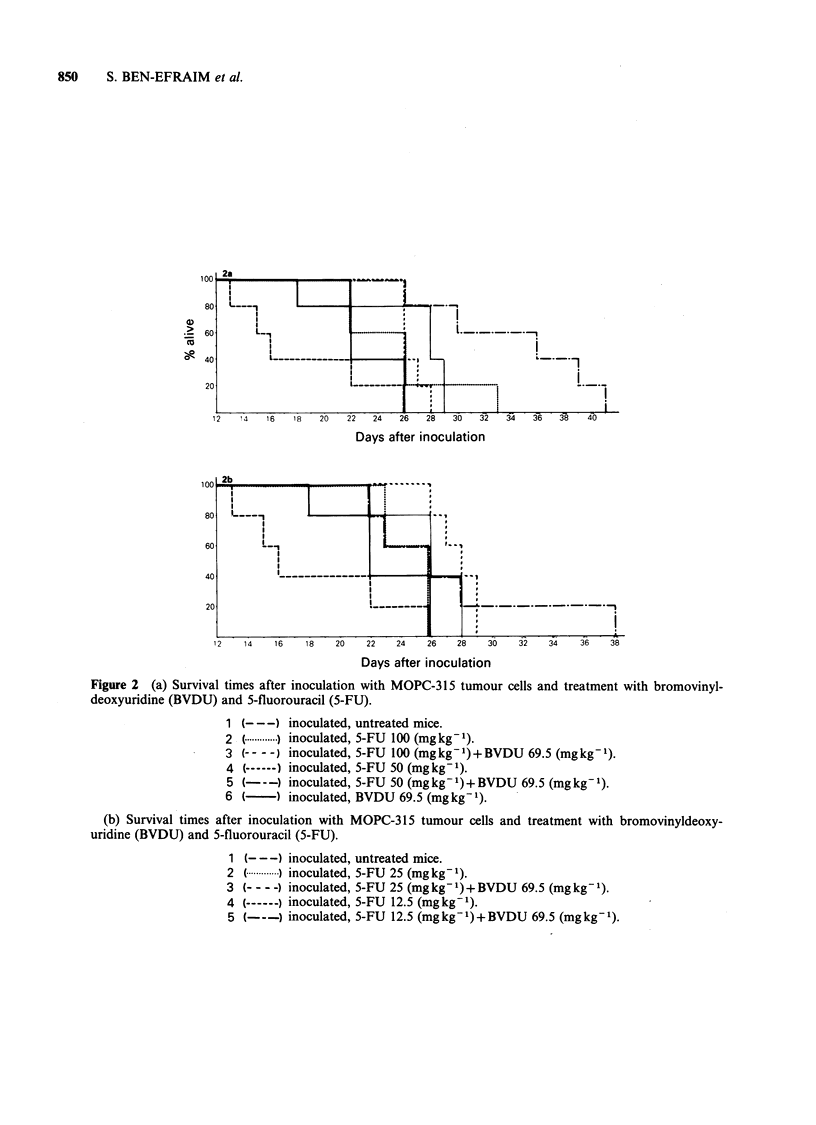

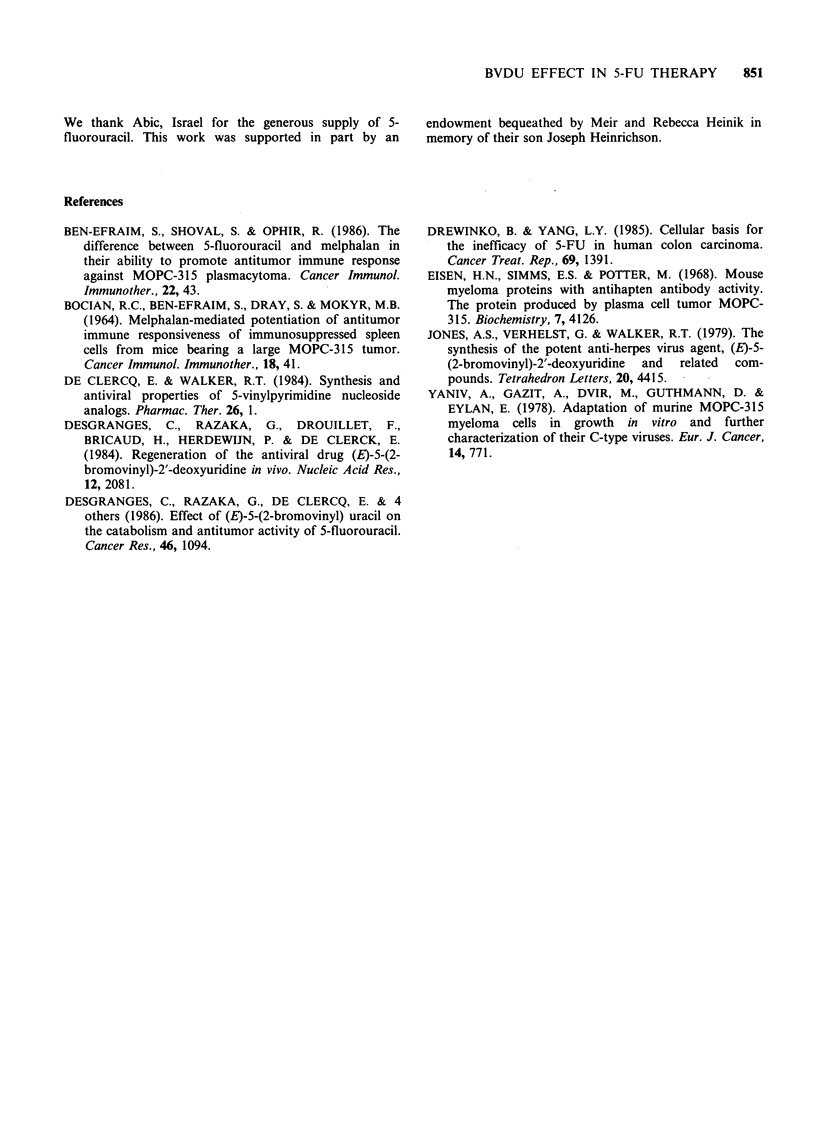


## References

[OCR_00386] Ben-Efraim S., Shoval S., Ophir R. (1986). The difference between 5-fluorouracil and melphalan in their ability to promote antitumor immune response against MOPC-315 plasmacytoma.. Cancer Immunol Immunother.

[OCR_00393] Bocian R. C., Ben-Efraim S., Dray S., Mokyr M. B. (1984). Melphalan-mediated potentiation of antitumor immune responsiveness of immunosuppressed spleen cells from mice bearing a large MOPC-315 tumor.. Cancer Immunol Immunother.

[OCR_00400] De Clercq E., Walker R. T. (1984). Synthesis and antiviral properties of 5-vinylpyrimidine nucleoside analogs.. Pharmacol Ther.

[OCR_00412] Desgranges C., Razaka G., De Clercq E., Herdewijn P., Balzarini J., Drouillet F., Bricaud H. (1986). Effect of (E)-5-(2-bromovinyl)uracil on the catabolism and antitumor activity of 5-fluorouracil in rats and leukemic mice.. Cancer Res.

[OCR_00405] Desgranges C., Razaka G., Drouillet F., Bricaud H., Herdewijn P., De Clercq E. (1984). Regeneration of the antiviral drug (E)-5-(2-bromovinyl)-2'-deoxyuridine in vivo.. Nucleic Acids Res.

[OCR_00418] Drewinko B., Yang L. Y. (1985). Cellular basis for the inefficacy of 5-FU in human colon carcinoma.. Cancer Treat Rep.

[OCR_00423] Eisen H. N., Simms E. S., Potter M. (1968). Mouse myeloma proteins with antihapten antibody acitivity. The protein produced by plasma cell tumor MOPC-315.. Biochemistry.

[OCR_00435] Yaniv A., Gazit A., Dvir M., Guthmann D., Eylan E. (1978). Adaptation of murine MOPC-315 myeloma cells to growth in vitro and further characterization of their C-type viruses.. Eur J Cancer.

